# Levels of Biosimilar Infliximab during and after Induction Treatment in Crohn’s Disease and Ulcerative Colitis—A Prospective Polish Population Study

**DOI:** 10.3390/jcm10225311

**Published:** 2021-11-15

**Authors:** Anna Pękala, Rafał Filip

**Affiliations:** 1 Department of Gastroenterology with IBD, Unit of Clinical Hospital 2 in Rzeszow, Lwowska 60, 35-301 Rzeszow, Poland; apekala@onet.pl; 2 Faculty of Medicine, University of Rzeszow, Aleja Majora Wacława Kopisto 2a, 35-210 Rzeszow, Poland

**Keywords:** CT-P13, biosimilar, anti-TNF, treatment response, treatment monitoring

## Abstract

Background: Primary lack or secondary loss of response to therapy with infliximab is a significant problem. This study aimed to evaluate the response to treatment in patients with Crohn’s disease (CD) and ulcerative colitis (UC) achieving therapeutic and sub-therapeutic trough levels of biosimilar infliximab (CT-P13). Results: A total of 65 patients (32 with CD and 33 with UC) were recruited. The overall response rate in both CD and UC patients exceeded 80%. There were no significant differences in treatment response and CT-P13 levels for patients with CD or UC. We did not find significant differences in the percentage of patients achieving drug levels of 3 μg/mL at week 6, 10, or 12; a significant decrease was observed at week 14. Up to 55.5% of patients with CD and 64.3% of patients with UC with sub-therapeutic CT-P13 levels at week 14 primarily responded to treatment. Conclusions: Intermediate measurements of drug levels at weeks 10 and 12 did not capture any pronounced decrease in infliximab concentrations below therapeutic levels in either group, thus suggesting no clinical usefulness. A significant percentage of patients primarily responded to treatment despite sub-therapeutic drug levels after the induction phase.

## 1. Introduction

Ulcerative colitis (UC) and Crohn’s disease (CD) are classified as immune-mediated, chronic inflammatory bowel diseases (IBD) [1]. Severe and moderate courses of these diseases, lack of response to standard treatment, and presence of poor prognostic factors are indications for use of biological drugs that inhibit chronic inflammatory response [2,3]. Tumor necrosis factor alpha (TNF-α) is one of the most important factors that maintain chronic inflammation. Three classes of biologics are available for the treatment of IBD: TNF inhibitors, adhesion molecule inhibitors, and anti-interleukin (IL) drugs. Among this type of drug, anti-IL-23 and anti-TNF alpha seem to secure the best results in the management of these conditions [4,5]. In addition to the original TNF inhibitors, biosimilar products are available [6]. Biosimilar infliximab (CT-P13) is a chimeric human–mouse monoclonal antibody against TNF-α. CT-P13 is frequently used as an effective and safe alternative for original infliximab (IFX) [7,8,9,10]. While it is an important drug in IBD treatment, a significant problem may be the primary lack or secondary loss of response (LOR) to therapy. Therefore, their prevention is an important subject addressed in clinical trials [11,12,13]. There is evidence to suggest that combination therapy or/and optimization of the dosing regimen can improve overall treatment efficacy [6,12].

Reactive monitoring of drug levels is a recommended practice in cases of decreased response or LOR. There are significant differences in drug requirements during maintenance treatment, as reflected by the dosage of 5 to 10 mg/kg every four to eight weeks [14,15]. The standard dosage of IFX in induction is 5 mg/kg; treatment monitoring during this period of therapy is not performed routinely and a therapeutic window has not been established for it [16]. In many studies, IFX levels were measured just before or after subsequent infusions of the drug at induction. However, little is known about the possible utility of intermediate measurements at weeks 10 and 12 and comparison between patients with CD and UC [17,18,19].

The aim of this study was to comparatively evaluate the response to treatment and the frequency of achieving and maintaining adequate (3 μg/mL) levels of CT-P13 during the induction phase at week 6 (before the third induction dose) and in the maintenance phase at weeks 10, 12, and 14 in patients with CD and UC. Furthermore, risk factors, the relationship between the level of drug and the response to induction therapy, as well as the occurrence of secondary LOR were analyzed.

## 2. Materials and Methods

### 2.1. Patients

Patients with CD and UC who qualified for the biosimilar infliximab (CT-P13, Remsima) [20] treatment within drug programs were consecutively enrolled between 2017 and 2019 at the tertiary IBD center in Rzeszow, Poland. The inclusion criterion for patients with CD was disease activity on the Crohn’s Disease Activity Index (CDAI) scale above 300 points or less, but with the presence of perianal fistulas. For patients with UC the inclusion crterion was disease activity above six points on the Mayo scale.

In both groups, CT-P13 was administered intravenously at a dose of 5 mg/kg according to the induction schedule at week 0, week 2, and week 6. In the maintenance phase, the drug was administered every four or eight weeks. The patients were monitored for 12 months.

The study was approved by the Ethics Committee of the University of Rzeszów (No 9 October 2016). Each participant read and signed an informed consent form.

### 2.2. CT-P13 Concentrations

**Figure 1:**
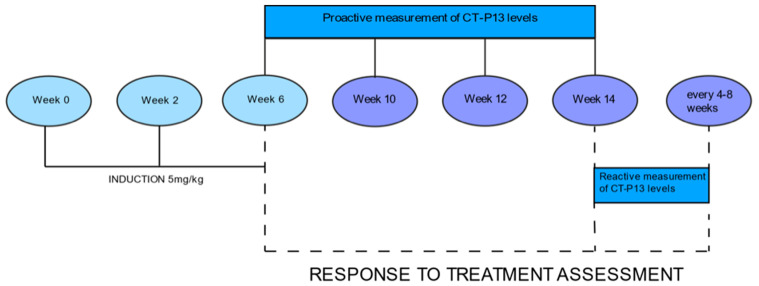
3 Response to treatment.

Drug levels were measured at week 6, just before the third induction dose, then at weeks 10, 12, and 14, just before the first maintenance dose (Figure 1). Serum levels of CT-P13 were evaluated using validated ELISA methods (Matriks Biotek, Ankara, Turkey). The detection range of the CT-P13 test was 0.4 μg/mL to 20 μg/mL, while the therapeutic range was established at 3 to 7 μg/mL [21]. We calculated the percentage of patients achieving CT-P13 levels equal to or greater than 3 μg/mL at each measurement.

Response to treatment was assessed at week 6, week 14, and then during maintenance treatment before each drug infusion (every four or eight weeks) for 12 months. In UC patients, the response to treatment was defined as reduction in disease activity by at least three points on the full Mayo scale and at least 30%, with decrease on the rectal bleeding subscale by at least one point [22]. In CD patients, the response to treatment was defined as reduction by at least 70 points and by at least 25% on the CDAI scale from baseline.

Primary lack of response in both groups was defined as none or little response, or deterioration after at least two infusions of infliximab. Response to treatment was analyzed at weeks 6 and 14 after three induction infusions of infliximab [23].

Secondary LOR was defined as deterioration after primary response to CT-P13 that resulted in terminating treatment or increasing dosage [24]. Response to treatment was assessed for all patients but was analyzed separately for patients who achieved drug levels of 3 μg/mL and for those with drug levels below 3 μg/mL at week 6. The same criteria were used for the assessment at week 14.

In addition, among patients with UC and CD, we analyzed the subgroups of patients who achieved the highest levels of CT-P13 (over the detection limit of 20 μg/mL) at week 6 and compared them with the patients who achieved lower drug levels.

### 2.3. Statistical Methods

Nominal data was presented by counts (*n*) and frequency of occurrence (%). Comparison of frequencies between groups was performed using the chi squared test. When group sizes did not allow for the use of the chi-squared test we performed Fisher’ s exact test. Furthermore, the ROC curves were prepared, and the optimal cut-off point for infliximab levels was determined as a diagnostic test for the response to treatment and the subsequent absence of secondary loss of response. The Youden index was used in determining the optimal cut-off point. All statistical tests were performed using R v. 4.0.5. software (The R Foundation for Statistical Computing, Wien, Austria) at a significance level of α = 0.05.

## 3. Results

### 3.1. Baseline Characteristics

The study group included 65 patients (32 with CD and 33 with UC). Patients with UC scored 7–12 points on the Mayo scale (moderate to severe disease) and CD activity ranged from 150 to 435 CDAI points. Most of the patients (86.2%) received concomitant treatment with thiopurines. Seventy-five percent of patients received additional mesalzine, and 55.4% of patients were also treated with glucocorticosteroids. Patient baseline characteristics are shown in Table 1.

### 3.2. Response to Treatment

Twenty-six of 32 (81.2%) CD patients responded to induction therapy. Primary lack of response was observed in six of 32 patients (18.8%). Loss of response was observed in 14 of 26 (53.8%) patients and could have been associated with insufficient drug levels. Subsequently, the dosage was increased to 5–10 mg/kg every four to eight weeks.

Twenty-seven of 33 (81.8%) UC patients responded to induction therapy. Primary lack of response was observed in six of 32 patients (18.2%). Loss of response was observed in 10 of 27 (37%) patients and was caused by non-therapeutic levels of infliximab (Table 2 and Table 3).

### 3.3. Levels of CT-P13

Additional measurements at weeks 10 and 12 did not capture a significant decrease in the percentage of patients achieving therapeutic drug concentrations compared to baseline (Table 2). A significant decrease was observed between weeks 6 and 14 in patients with UC (*p* = 0.014). In patients with CD, drug concentrations of 3 μg/mL were observed in 84.4% of patients at week 6, in 84.0% at week 10, in 80.0% at week 12, and in 68.7% at week 14 (Table 2). In the UC subgroup, drug levels of 3 μg/mL were found in 84.8% of patients at week 6, in 88.5% at weeks 10 and 12, and in 57.6% at week 14.

### 3.4. Comparative Evaluation for Patients with Crohn’s Disease and Ulcerative Colitis

#### 3.4.1. Response to Treatment

A comparable number of patients with CD and UC (81.2% vs. 81.8%) achieved a response to induction (*p* > 0.999). A similar number of patients with CD (84.4%) and UC (84.8%) achieved trough levels of CT-P13 of 3 μg/mL at week 6 (*p* > 0.999). Some differences, although not statistically significant, were observed at week 14 when the therapeutic levels of CT-P13 were found in 68.8% patients with CD and in 57.6% patients with UC (*p* = 0.499) (Table 3).

In the CD subgroup, response to treatment was observed in 88.9% of patients who achieved CT-P13 levels of 3 μg/mL at week 6 and in 40% of patients whose drug levels were lower (*p* = 0.034). At week 14, response to treatment was found in 90.9% of patients with therapeutic drug levels and in 55.5% of patients with non-therapeutic levels of CT-P13 (*p* = 0.043).

In patients with UC, response to treatment was observed in 92.8% of patients with levels of CT-P13 equal to or above 3 μg/mL and in 20% of patients who did not achieve the trough drug level of 3 μg/mL at week 6 (*p* = 0.002). At week 14, response to treatment was found in 94.7% of patients with therapeutic drug levels and in 64.3% of patients with non-therapeutic levels of CT-P13 (*p* = 0.062) (Table 3). Analysis showed a statistically significant association between minimum therapeutic drug levels at week 6 and response to treatment in patients with CD and UC. At week 14, the significant relationship was found only in patients with CD.

**Figure 2:**
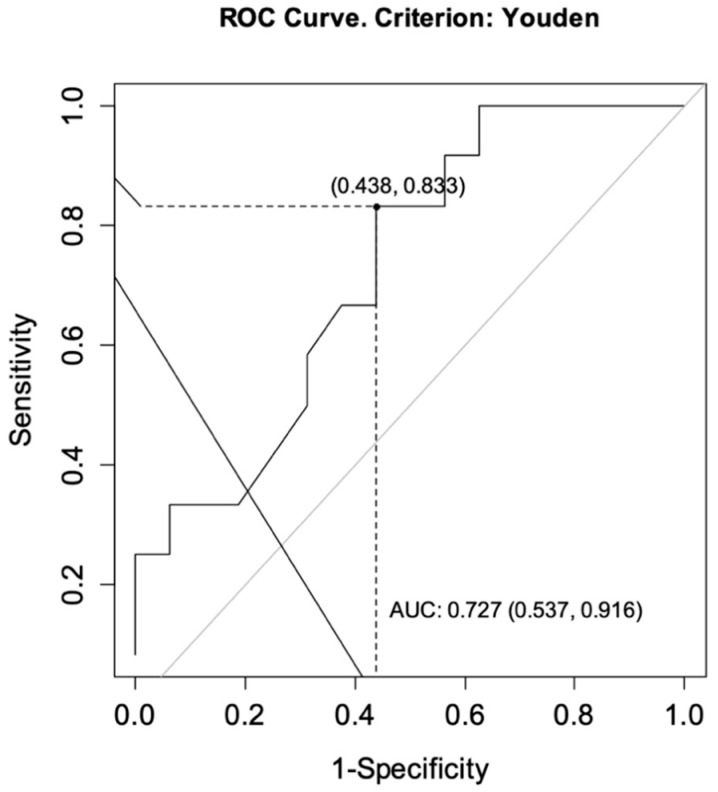
ROC curve for infliximab levels as a predictor of achieving a primary response while ensuring no secondary loss of response in patients with Crohn’s disease. AUC, area under the curve.

**Figure 3:**
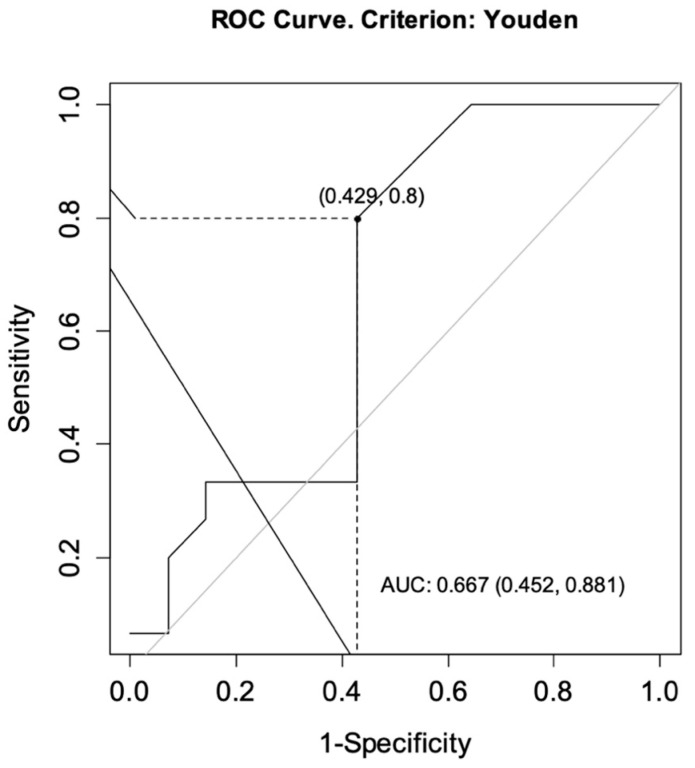
ROC curve for infliximab levels as a predictor of achieving a primary response while ensuring no secondary loss of response in patients with ulcerative colitis. AUC, area under the curve.

The ROC curves were drawn for each group to determine the optimal cut-off points for CT-P13 level as predictors of achieving a primary response while ensuring no secondary loss of response. For CD, the analysis showed an area under the curve (AUC) = 0.727, CI95 [0.537; 0.916] indicating satisfactory differentiation of patients by the selected diagnostic test. The optimal cut-off point for infliximab at week 14 was calculated to be 4.60 μg/mL. The sensitivity of the analysis was 83%, specificity 56%, and test accuracy 68% (Figure 2). For UC patients, the same analysis showed AUC = 0.667, CI95 [0.452; 0.881] and the optimal cut-off point for CT-P13 at week 14 was calculated to be 3.10 μg/mL. The sensitivity of the analysis was 80%, specificity 57%, and test accuracy 69% (Figure 3).

#### 3.4.2. Secondary Loss of Response

We observed a higher incidence of LOR in patients with CD (53.8%) compared to UC (37.0%). The difference between groups was not statistically significant (*p* = 0.341). Patients with CD who achieved the minimum therapeutical levels of CT-P13 at week 14 lost response more often than those with UC (45.0% vs. 27.8%), but again, the difference was not statistically significant (*p* = 0.272). Among patients who did not reach the therapeutic minimum of the drug at week 14, all the patients with CD versus 55.5% of patients with UC lost their response to infliximab treatment. The differences were not statistically significant (*p* = 0.272) (Table 3).

#### 3.4.3. Risk Factors

A higher incidence of primary non-response or LOR was not confirmed for any of the potential risk factors (sex, lack of thiopurine use, history of infliximab treatment) (Table 4 and Table 5).

#### 3.4.4. Highest CT-P13 Levels

An additional focus of the analysis was to evaluate patients with the highest CT-P13 levels (>20 μg/mL, above the assay detection level) found at weeks 6 and 10 in both groups for any advantage in achieving a response to induction therapy compared to the other patients. The results are shown in Table 6.

In both groups, similar numbers of patients achieved CT-P13 levels greater than 20 μg/mL at weeks 6 and 10 (43.7% in CD and 39.4% in UC). In these patients, a high induction response rate was achieved (85.8% in CD and 92.3% in UC). The results were compared with a 66.6% response rate in CD and a 70% response rate in UC for patients who had lower CT-P13 levels at weeks 6 and 10, but the difference was not statistically significant. No association was confirmed between drug levels greater than 20 μg/mL at weeks 6 and 10 and response to induction (*p* = 0.412 in CD, *p* = 0.202 in UC). All patients with CD and only 76.9% of patients with UC who achieved CT-P13 levels above 20 μg/mL at weeks 6 and 10 maintained minimal therapeutic drug levels at week 14 (*p* = 0.098). There was also a high rate of LOR among patients with the highest drug levels: 50% in CD and 25% in UC (*p* = 0.400).

## 4. Discussion

Monitoring of biologic therapy with anti-TNF-α drugs can be proactive or reactive and is most often performed during the maintenance phase to optimize therapy. The benefits of reactive monitoring during maintenance treatment have been demonstrated [24,25]. Proactive monitoring and monitoring during induction analyzed in studies give different and sometimes contradictory results [26,27,28,29]. Many studies show that IFX levels in adult and pediatric patients responding to induction are significantly higher than in non-responding patients [30,31,32,33].

We found similar results in our previous study where the mean drug concentration in responders was 16.7 μg/mL, while in the group with no response it was 0.95 μg/mL [34]. In the current study, we attempted to determine the drug level that was associated with clinical response and with no LOR during one year of treatment. We identified a cut-off point for biosimilar IFX at week 14 of 4.6 μg/mL for CD and of 3.1 μg/mL for UC. Post-hoc analysis of the ACCENT I trial showed that sustained clinical response in patients with CD was associated with IFX levels equal to or greater than 3.5 μg/mL at week 14 [35]. A much higher IFX level of 7 µg/mL was found to be optimal and associated with remission at weeks 14 and 54 in the British PANTS trial, which included patients with CD treated with original and biosimilar IFX [31]. For UC patients, mucosal healing was associated with infliximab concentrations ≥5.1 μg/mL at week 14 and ≥2.3 μg/mL at week 30. Endoscopic remission was observed with IFX concentrations ≥6.7 μg/mL at week 14 and ≥3.8 μg/mL at week 30 [36]. These differences seem to suggest that the optimal level of IFX to ensure response or remission may be highly individual and dependent on many factors, including disease severity, as demonstrated in a group of children with UC treated at a dose of 5 or 10 mg/kg in induction [37].

However, recent studies show that monitoring of treatment at induction and dose selection based on drug concentration to achieve appropriately high post-induction levels of IFX may not be beneficial in IBD or other autoimmune diseases [38]. In our study, we also assessed a group of patients who had the highest (over the test detection level of 20 µg/mL) CT-P13 concentrations at week 6 and 10 and we compared them with the rest of the patients. Twenty-seven of 65 patients had the highest drug levels; however, we did not observe a statistically higher number of patients experiencing primary response nor decreased risk LOR in this subgroup. In patients with UC, the highest CT-P13 levels before last induction dose and four weeks later resulted in therapeutic drug levels (3 μg/mL) at week 14 (before the first maintenance dose) in only 75% of patients.

Despite these results, the data we analyzed on biosimilar IFX levels in association with treatment response support the benefit of proactive monitoring. The results suggest that proactive monitoring at weeks 6 and 14 provides important information because, as our previous study showed, indeterminate levels of biosimilar IFX at week 6 were associated with a high risk of immunization and nonresponse to therapy [34]. These results are in line with other research [34,39]. As for the intermediate measurements at weeks 10 and 12, our data did not confirm their usefulness in either CD or UC because IFX concentrations in more than 80% of patients were still within therapeutic limits. A significant deficiency of the drug was seen only at week 14; nevertheless, a decreasing trend could be detected at week 12 in some patients.

An interesting observation that suggests the utility of proactive monitoring is that primary response was achieved by more than 81% of patients in both groups but much fewer patients, only 68.8% of CD patients and 57.6% of UC patients, achieved the minimum therapeutic levels of CT-P13 at the end of induction. This shows that a considerable group of patients responded to induction treatment despite non-therapeutic drug levels. This was more evident in the UC group, as the non-therapeutic drug levels at week 14 were shown to be unrelated to non-response. This might have been caused by the influence of corticosteroids, which were used in more than 60% of UC patients at the start of treatment and may have masked both clinical and endoscopic response. At follow-up, all patients with CD and more than half of the patients with UC in this subgroup had lost response to treatment. This suggests that patients with non-therapeutic drug levels at week 14 require further monitoring and surveillance as the risk of LOR is significant in these patients. In our study, this was particularly evident in the CD group.

A limitation of this study is a relatively small patient group, which means that statistical differences between subgroups were not apparent. It would probably be worth continuing similar studies on a larger group of patients with IBD to verify the initial findings.

## 5. Conclusions

There is an unmet clinical need to establish IFX values predictive of treatment response for early identification of the subgroup of IBD patients that have the highest probability of non-response for potential IFX dosage adjustment. In our study, we have shown that overall response rate in both CD and UC exceeded 80% and there were no significant differences in primary response and biosimilar IFX levels across measurement points for both CD and UC groups. Intermediate measurements of drug levels at weeks 10 and 12 did not capture any pronounced decrease in IFX concentrations below therapeutic levels in either group.

The study also showed that the group of patients who achieved the highest levels of CT-P13 before the third induction dose was not significantly different from the other patients in terms of response to induction and LOR. A significant number of patients who achieved a primary response presented subtherapeutic levels of CT-P13 at week 14.

## Figures and Tables

**Table 1:** Patient baseline characteristics.

	Crohn’s Disease Patients	Ulcerative Colitis Patients
*n*	32	33
Females, *n* (%)	17 (53.1)	14 (42.4)
Males, *n* (%)	15 (46.9)	19 (57.6)
Age, median (range) years	30.6 (21–54)	38.9 (20–81)
Disease duration (IQR) years	3.4 (0.5–13)	5.2 (0.5–11)
Smoking status, *n* (%)		
Never smoked	27 (84.4)	30 (90.9)
Ex-smoker	3 (9.4)	2 (6.0)
Current smoker	2 (6.2)	1 (3.1)
Concomitant treatment, *n* (%)		
Thiopurines	26 (81.2)	30 (90.9)
Steroids	15 (46.80)	21 (63.6)
Mesalazine	16 (50.0)	33 (100.0)
**Crohn’s disease**		
Age at diagnosis, *n* (%)	A1 (diagnosed <17 years of age)	4 (12.5)
	A2 (diagnosed 17–40 years of age)	29 (90.6)
	A3 (diagnosed >40 years of age)	0
Disease location, *n* (%)	L1(ileal)	6 (18.7)
	L2 (colonic)	7 (21.8)
	L3 (ileocolonic)	18 (56.2)
	L3 + (ileocolonic) ++ L4 (upper gastrointestinal tract)	1 (3.1)
Disease behavior, *n* (%)	B1 (nonstricturing, nonpenetrating)	22 (68.7)
	B2 (stricturing)	3 (9.4)
	B3 (penetrating)	7 (21.9)
CDAI indicator at the start of treatment, mean (range)	266.6 (150–435)	
**Ulcerative colitis**		
Extent, *n* (%)	E1 (proctitis)	2 (6.0)
	E2 (left-sided colitis)	21 (63.6)
	E3 (pancolitis)	10 (30.3)
Severity, *n* (%)	S1 (mild)	5 (15.2)
	S2 (moderate)	20 (60.6)
	S3 (severe)	8 (24.2)
Mayo score at the start of treatment, mean (range)	8.3 (7–12)	

**Table 2:** Number of patients with CD and UC who achieved CT-P13 level of 3 μg/mL.

	Week 6	Week 10	Week 12	Week 14
Crohn’s disease	84.4%	84.0%	80.0%	68.7%
Ulcerative colitis	84.8%	88.5%	88.5%	57.6%

**Table 3:** Comparison of treatment response in both groups according to CT-P13 levels at weeks 6 and 14.

	Crohn’s Disease	Ulcerative Colitis	*p*-Value
*n*	32	33	
Response to treatment (in total)	26/32 (81.2%)	27/33 (81.8%)	>0.999
Primary lack of response (in total)	6/32 (18.8%)	6/33 (18.2%)	
Secondary loss of response (in total)	14/26 (53.8%)	10/27 (37.0%)	0.341
	**Week 6**		
Patients who achieved CT-P13 levels of 3 μg/mL at week 6	27/32 (84.4%)	28/33 (84.8%)	>0.999
Primary response	24/27 (88.9%)	26/28 (92.8%)	0.670
Primary lack of response	3/27 (11.1%)	2/28 (7.2%)	
Secondary loss of response	12/24 (50%)	5/26 (19.2%)	0.046
Patients who did not achieve CT-P13 levels of 3 μg/mL at week 6	5/32 (15.6%)	5/33 (15.1%)	>0.999
Primary response	2/5 (40.0%)	1/5 (20.0%)	>0.999
Primary lack of response	3/5 (60.0%)	4/5 (80.0%)	
Secondary loss of response	2/2 (100%)	0/1 (0%)	0.333
	**Week 14**		
Patients who achieved therapeutic CT-P13 levels of 3 μg/mL at week 14	23/32 (71.9%)	19/33 (57.6%)	0.499
Primary response	21/23 (91.3%)	18/19 (94.7%)	>0.999
Primary lack of response	2/23 (8.7%)	1/19 (5.3%)	
Secondary loss of response	9/21 (42.8%)	5/18 (27.8%)	0.272
Patients who did not achieve therapeutic CT-P13 levels of 3 μg/mL at week 14	9/32 (28.1%)	14/33 (42.4%)	0.344
Primary response	5/9 (55.5%)	9/14 (64.3%)	>0.999
Primary lack of response	4/9 (44.4%)	5/14 (35.7%)	
Secondary loss of response	5/5 (100%)	5/9 (55.5%)	0.221

Data is presented as number of subjects (% of group) and frequency comparison using chi-squared test or Fisher’ s exact test.

**Table 4:** Primary lack of response.

	Crohn’s Disease (*n* = 32)			Ulcerative Colitis (*n* = 33)		
	Primary Lack of Response	Response to Treatment	*p*	Primary Lack of Response	Response to Treatment	*p*
	*n* = 6	*n* = 26		*n* = 6	*n* = 27	
Sex						
Women	3 (50.0%)	14 (53.8%)	>0.999	3 (50.0%)	11 (40.7%)	>0.999
Men	3 (50.0%)	12 (46.1%)		3 (50.0%)	16 (59.3%)	
Concomitant treatment with thiopurines						
Yes	5 (83.3%)	21 (80.8%)	>0.999	6 (100%)	24 (88.9%)	>0.999
No	1 (16.7%)	5 (19.2%)		0 (0%)	3 (11.1%)	
History of treatment with infliximab						
Yes	1 (16.7%)	2 (7.6%)	0.476	0 (0%)	2 (7.4%)	>0.999
No	5 (83.3%)	24 (92.3%)		6 (100%)	25 (92.6%)	

Data is presented as number (%) of subjects and frequency comparison using chi-squared test or Fisher’ s exact test.

**Table 5:** Secondary loss of response.

	Crohn’s Disease (*n* = 32)			Ulcerative Colitis (*n* = 33)		
	Secondary Loss of Response	Response to Treatment	*p*	Secondary Loss of Response	Response to Treatment	*p*
	*n* = 14	*n* = 18		*n* = 10	*n* = 23	
Sex						
Women	9 (64.3%)	8 (44.4%)	0.265	5 (50.0%)	9 (39.1%)	0.562
Men	5 (35.7%)	10 (55.6%)		5 (50.0%)	14 (60.9%)	
Concomitant treatment with thiopurines						
Yes	13 (92.9%)	13 (72.2%)	0.196	10 (100%)	20 (87.0%)	0.536
No	1 (7.1%)	5 (27.8%)		0 (0%)	3 (13.0%)	
History of treatment with infliximab						
Yes	1 (7.1%)	2 (11.1%)	>0.999	1 (10.0%)	1 (4.3%)	0.521
No	13 (92.9%)	16 (88.9%)		9 (90.0%)	22 (95.6%)	

Data is presented as number (%) of subjects and frequency comparison using chi-squared test or Fisher’ s exact test.

**Table 6:** CT-P13 levels >20 μg/mL and < 20 μg/mL at weeks 6 and 10 in patients with CD and UC and response to treatment.

	Crohn’s Disease			Ulcerative Colitis		
	Level >20 μg/mL at Weeks 6 and 10	Level <20 μg/mL at Weeks 6 and 10	*p*	Level >20 μg/mL at Weeks 6 and 10	Level <20 μg/mL at Weeks 6 and 10	*p*
	*n* = 14	*n* = 18		*n* = 13	*n* = 20	
Response to treatment	12/14 (85.8%)	12/18 (66.6%)	0.412	12/13 (92.3%)	14/20 (70.0%)	0.202
Therapeutic levels of CT-P13 at week 14	12/12 (100%)	9/12 (75.0%)	0.217	9/12 (75.0%)	8/14 (57.1%)	0.429
Secondary loss of response	6/12 (50.0%)	8/12 (66.6%)	0.680	3/12 (25.0%)	7/14 (50.0%)	0.248
Lack of response	2/14 (14.2%)	6/18 (33.4%)	0.412	1/13 (7.7%)	6/20 (30.0%)	0.202
Therapeutic levels of CT-P13 at week 14	2/2 (100%)	0/6 (0%)	0.036	1/1 (100%)	1/6 (16.7%)	0.286

Data is presented as number of subjects (% of group) and frequency comparison using chi-squared test or Fisher’ s exact test.

## Data Availability

The datasets generated during and analysed during the current study are available from the corresponding author on reasonable request.
